# Evaluation of a magnetic resonance-compatible dentoalveolar tactile stimulus device

**DOI:** 10.1186/1471-2202-11-142

**Published:** 2010-10-28

**Authors:** Estephan J Moana-Filho, Donald R Nixdorf, David A Bereiter, Mike T John, Noam Harel

**Affiliations:** 1 Center for Neurosensory Disorders, University of North Carolina, Chapel Hill, NC, USA; 2 Division of TMD & Orofacial Pain, University of Minnesota, Minneapolis, MN, USA; 3 Department of Neurology, University of Minnesota, Minneapolis, MN, USA; 4 Department of Diagnostic & Biological Sciences, University of Minnesota, Minneapolis, MN, USA; 5 Division of Epidemiology & Community Health, University of Minnesota, Minneapolis, MN, USA; 6 Center for Magnetic Resonance Research, University of Minnesota, Minneapolis, MN, USA

## Abstract

**Abstract:**

**Background**

Few methods exist to study central nervous system processes following dentoalveolar tactile stimulation using functional magnetic resonance imaging (fMRI), likely due to inherent technical difficulties. Our primary goal was to develop and perform feasibility testing of a novel device capable of delivering valid and reliable dentoalveolar stimuli at dental chair-side and during MRI. Details of a device designed to deliver dentoalveolar dynamic pressure stimuli are described. Device testing took place in three settings: a) laboratory testing to assess range of stimulus force intensities, b) dental chair-side to assess reliability, validity and discriminant ability in force-pain relationship; and c) MRI to evaluate magnetic compatibility and ability to evoke brain activation in painfree subjects similar to those described in the literature.

**Results**

A novel device capable of delivering valid and reliable dentoalveolar somatosensory stimulation was developed (ICC = 0.89, 0.78-1 [95% CI]). Psychophysical data analysis showed high discriminant ability in differentiating painfree controls from cases with chronic dentoalveolar pain related to deafferenting dental procedures (sensitivity = 100%, specificity = 86.7%, area under ROC curve = 0.99). FMRI results of dentoalveolar dynamic pressure pain in painfree subjects revealed activation of brain areas typically associated with acute pain processing including thalamus, primary/secondary somatosensory, insular and prefrontal cortex.

**Conclusions**

A novel psychophysical method to deliver dynamic dentoalveolar pressure stimulation was developed and validated, allowing non-invasive MRI-based exploration of central nervous system function in response to intraoral somatosensation.

**Background:**

The organization of the trigeminal system is unique as it provides somatosensory innervation to the face, masticatory and oral structures, the majority of the intracranial contents [1] and to specialized structures (tongue, nasal mucosa, auricle, tympanic membrane, cornea and part of the conjunctiva) [2]. Somatic sensory information transmitted by the trigeminal nerve is crucial for normal orofacial function; however, the mechanisms of many chronic pain conditions affecting areas innervated by this sensory system are not well understood [3-5]. The clinical presentation of chronic intraoral pain in the area of a tooth or in a site formally occupied by a tooth with no clinical or radiological signs of pathology, referred to as atypical odontalgia (AO) [6,7], is one such chronic pain condition of particular interest to dentists that is difficult to diagnose and manage. Recent research suggests both peripheral and central nervous system mechanisms being involved in AO pathophysiology [8-10], but the majority of mechanism-based research of patients with AO has focused on the "peripheral aspect" [7].

Functional magnetic resonance imaging (fMRI) is an established research technique to study the central aspects of pain [11]. Of existing neuroimaging techniques, fMRI provides good spatial resolution of cortical and subcortical structures critical in the processing of nociception, acceptable temporal resolution, does not involve ionizing radiation, and can be performed using most MRI systems that already exist in research centers and the community. For these reasons, we sought to develop a protocol that allows us to use this tool to investigate the central mechanisms involved in the processes of intraoral pain arising from the dentoalveolar region. Using this device, our long-term objective is to improve our understanding of the underlying mechanisms of persistent dentoalveolar pain.

In the past few years several studies used fMRI to investigate the human trigeminal system [12,13], with a limited subset focusing on intraoral stimulation - specifically on the dentoalveolar processes, such as lip, tongue and teeth stimulation [14] or only teeth [15-17]. Some reasons for scarce literature on this topic may be the technical challenges involved in delivering facial/intraoral stimulation inside a MR scanner [17,18]: possibility of magnetic interference, detriment of image quality, subject discomfort and reduced working space between the subject's head and the radiofrequency coil. As a consequence a MR-compatible device would need to not only overcome these challenges but also be capable of delivering a controlled and reproducible stimuli [19], as reliability/reproducibility is a necessary feature of sensory testing [20].

Existing MR-compatible methods of dentoalveolar stimulation are limited and do not adequately deliver stimuli across a range of non-painful to painful intensities and/or cannot be adjusted to reach posterior aspects of the dentoalveolar region. Therefore our goal was to develop and test the feasibility of a device able to: 1) provide reliable and valid dentoalveolar stimuli, 2) deliver such stimulation within the restricted space of an MR head coil, 3) be compatible for use within an MR environment, and 4) produce brain activation in painfree controls consistent to those observed by others using fMRI.

## Results

### Subjects

Five painfree female control subjects were enrolled and completed the study protocol, as well as five age- and gender-matched AO patients were used for threshold testing only. Their summary data is presented in Table 1.

**Table 1:** Study participant characteristics

Subjects group	Age (yrs)	**Handedness**^1^	Stimulus location	Pressure threshold	Pain intensity
Painfree			(quadrant/closest tooth)	(average # elastic bands)	(0-10 numerical scale)
1	54	R	Right Upper/#5	5 (± 1)	4*
2	52	R	Left Upper/#13	8 (± 1)	0*
3	50	L	Right Upper/#5	7 (± 0)	4*
4	48	R	Left Upper/#13	6 (± 0)	5**
5	52	R	Right Upper/#3	5 (± 0)	3*

**Average:**	**51 (± 2)**			**6 (± 1)**	**3**
Atypical Odontalgia					

6	57	R	Right Upper/#3	2 (± 1)	4**
7	59	R	Right Upper/#5	2 (± 1)	5**
8	49	R	Left Lower/#19	1 (± 1)	3.5**
9	47	L	Left Upper/#14	4 (± 1)	4**
10	56	R	Right Upper/#3	2 (± 1)	4**
**Average:**	**53 (± 5)**			**2 (± 0)**	**4**

Self-report. *Pain rating at the end of fMRI session. **Pain rating at 1-week threshold.

Parentheses indicate standard deviation.

### Intraoral stimulus device

**Figure 1:**
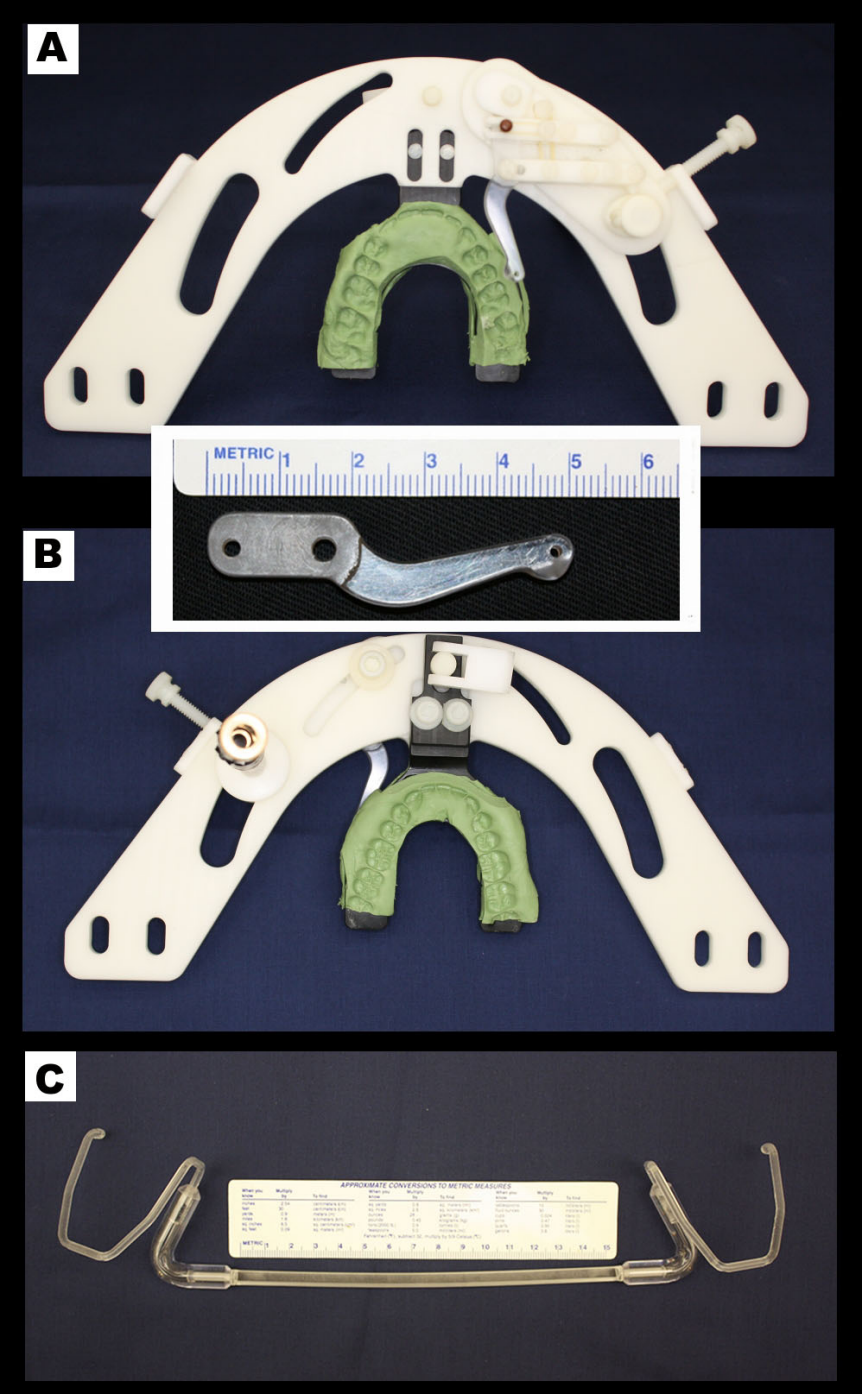
**Stimulus device**. Superior (A) and inferior (B) views of the stimulator device (inset: close view of intraoral probe), (C) image of lip and cheek retractor.

The stimulus device is depicted in Figure 1(A, B) fully assembled, and an approximate view of the intraoral probe shows its design that allows contact to the desired dentoalveolar location and avoids touching the labial commissure.

#### Laboratory force measurements

Resistance readings from the strain gauge attached to the intraoral probe and the amount of weight placed at the probe tip showed a linear relationship up to a "failure" point, i.e., when the probe translated. For each 3.175 mm (1/8") elastic band a weight was needed to elicit such translation (1 = 70 g; 2 = 150 g; 3 = 170 g; 4 = 220 g; 5 = 290 g; 6 = 370 g; 7 = 400 g; 8 = 440 g). This laboratory test gave an estimate of the force delivered by the intraoral probe tip with different numbers of 3.175 mm (1/8") elastic bands and provided evidence that the device exerted a near linear response with increasing numbers of elastic bands.

#### Psychophysical data

Dynamic pressure pain thresholds at the three time points (Table 2) showed strong agreement (ICC = 0.89; 95% CI 0.78-1.00). One-way random effects ANOVA provided a receiver operating characteristic (ROC) curve, with the area under this curve describing the device discriminant ability. From the cutoff points derived from the forces delivered by the intraoral probe, it was determined that when using 4 elastic bands (≈220 g) AO subjects and controls could be differentiated with sensitivity of 100%, specificity of 86.7% with an area under the ROC curve = 0.99.

**Table 2:** Individual dynamic pressure pain thresholds

	Initial	1-hour	1-week	Mean (std deviation)
Painfree				
1	4	4	6	5 (± 1)
2	8	7	8	8 (± 1)
3	7	7	7	7 (± 0)
4	6	6	6	6 (± 0)
5	5	5	5	5 (± 0)

Atypical Odontalgia				

6	2	3	2	2 (± 1)
7	3	3	1	2 (± 1)
8	1	1	2	1 (± 1)
9	4	4	3	4 (± 1)
10	1	2	2	2 (± 1)

Values represent the number of 3.175 mm (1/8") elastic bands used to produce pressure

Pain ratings collected during blocked-design stimulation at dental chair-side during the full stimulus protocol (second visit) had considerable intra- and inter-subjects variation. Two controls did not report pain during stimulation despite use of 8 elastic bands, while the other 3 controls rated pain within the target range (3 to 5 out of 10). Two AO subjects on the other hand reported pain levels above the target range, even though they were stimulated using same forces as the 1-week pressure pain threshold established at the beginning of the second visit.

#### Functional Magnetic Resonance Imaging data

**Figure 2:**
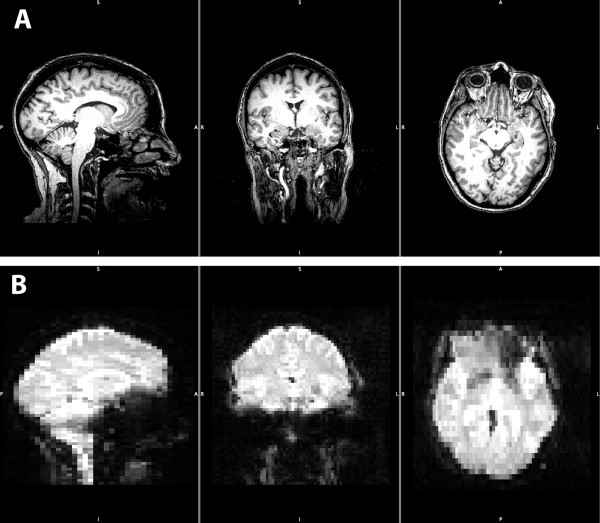
**Example anatomical and functional images**. (A) displays example anatomical images of a subject's head scan before pre-processing for statistical analysis. Anatomical images have a resolution of 1 mm isotropic voxels and were taken with the stimulus device in place intraorally. (B) displays example functional MR images (a.k.a. EPI) of the same subject before pre-processing. These functional images have a resolution of 3 mm by 3 mm in plane (sagital, coronal) and 5.625 mm out of plane (axial). Minimal distortion under visual inspection is present in these images, except for the loss of signal (a.k.a. drop-out due to the air-tissue interface) around the frontal lobes in the lower right panel. In both (A) and (B), from left to right, images are mid sagittal, coronal, and axial in orientation.

**Figure 3:**
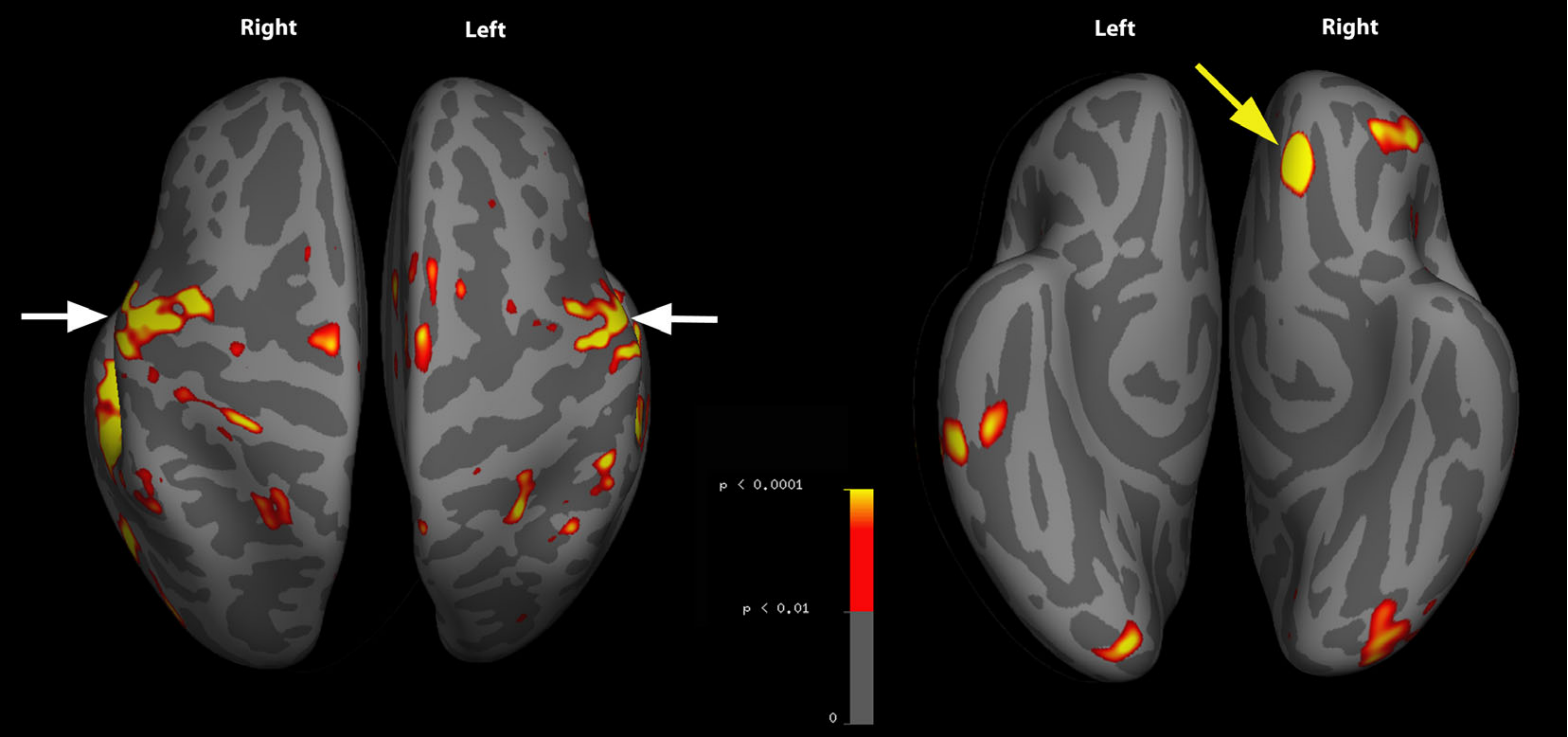
**Inflated superior and inferior cortical surface activity**. Aggregate data from all 5 subjects with arrows pointing to primary somatosensory (white) and prefrontal (yellow) cortices. Gyri = light grey; sulci = dark grey.

**Figure 4:**
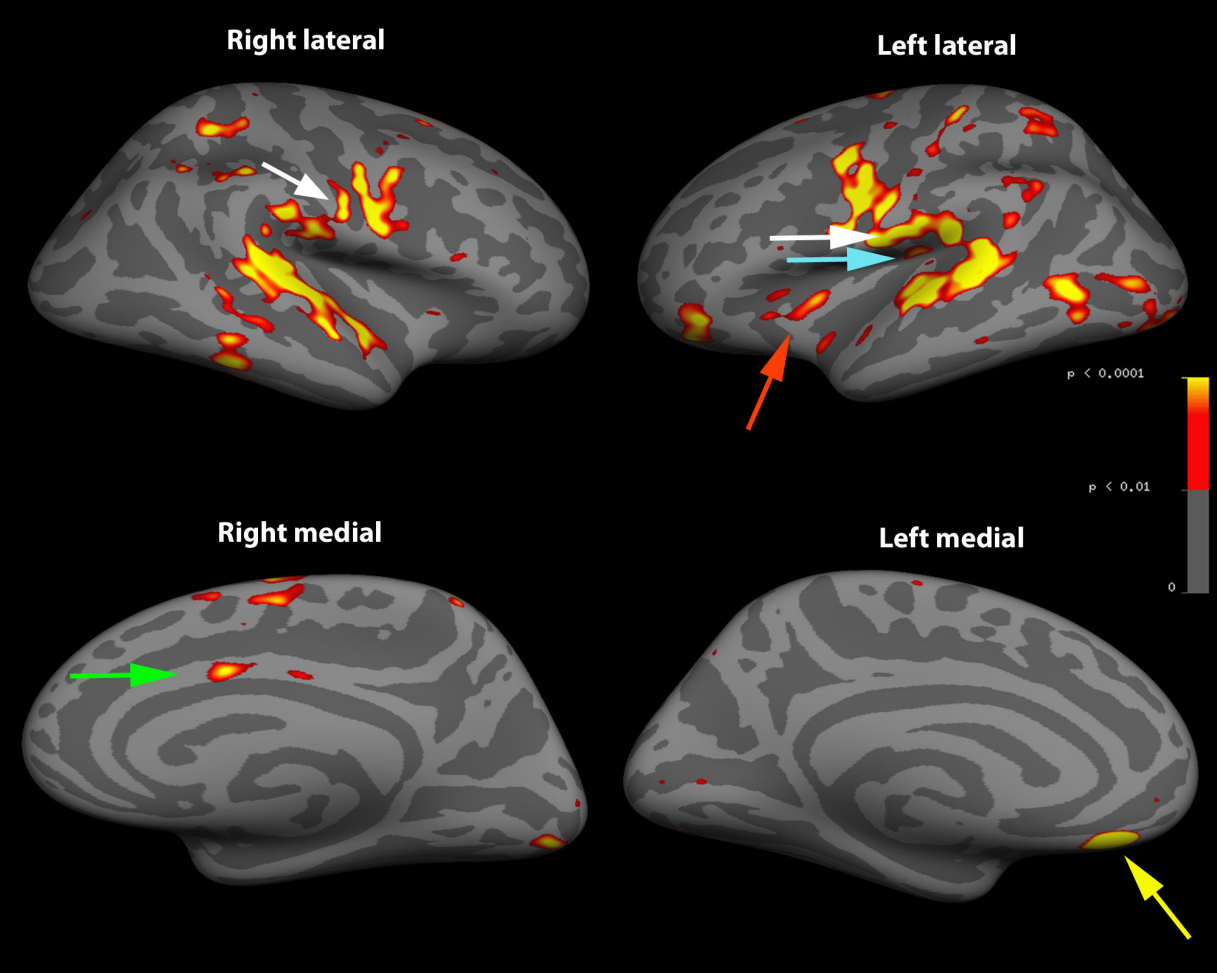
**Inflated lateral and medial cortical surface activity**. Aggregate data from all 5 subjects with arrows pointing to primary (white) and secondary (blue) somatosensory, prefrontal (yellow), anterior cingulate (green) and insular (red) cortices. Gyri = light grey; sulci = dark grey.

**Figure 5:**
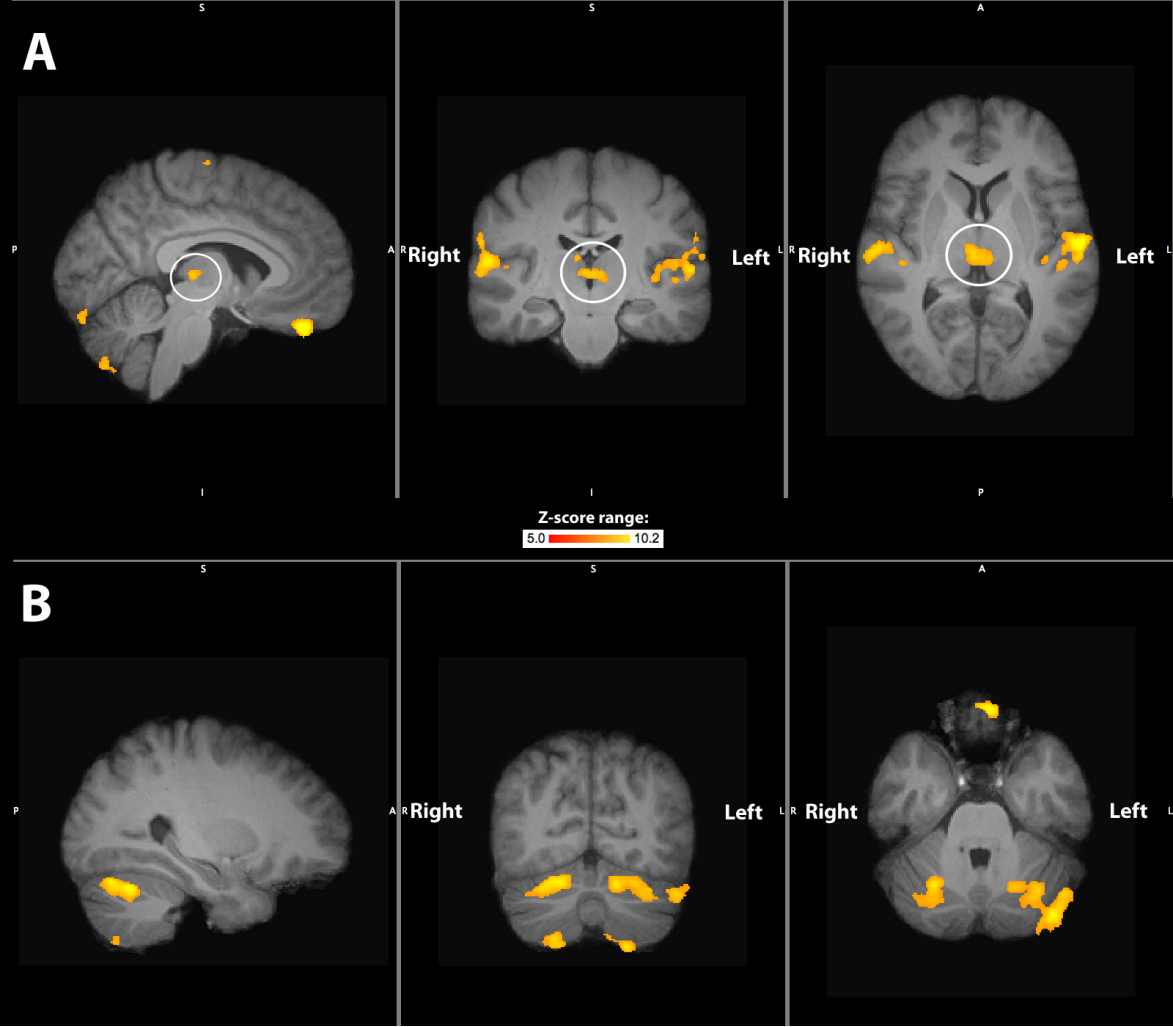
**Subcortical brain activations**. Row (A) depicts thalamus activity (in white circle) and row (B) depicts cerebellum activity from all 5 subjects. Left panels are sagittal sections, while the middle panels are coronal and the right panels axial sections.

FMRI data was collected from painfree controls only. Visual inspection of structural and functional MR images from all subjects detected minimal distortion, despite the presence of the stimulus device within the imaging field of view during the whole session (Figure 2). Head motion was minimized as a result of bite bar use (mean peak displacement during functional imaging = 0.37 mm (95%CI 0.26-0.49)). FSL group-level results revealed that dentoalveolar dynamic pressure pain activated several brain regions including primary & secondary somatosensory, prefrontal, anterior cingulate, and insular cortices, as well as the thalamus and cerebellum (Table 3). These findings are consistent with what others have observed and are considered part of a network for acute pain in painfree controls [21]. Images of the activation maps from cortical surface-based group analysis using Freesurfer are displayed in Figure 3 and 4, and subcortical activations from FSL group analysis overlaid on the group mean structural image are shown in Figure 5.

**Table 3:** Group-level brain activations

	MNI Coordinates						
**Brain region**	**X**	**Y**	**Z**	**Maximum** **Z-value**	**Cluster size** **(mm^3^)**	**Cluster P-** **value**	**Acute pain** **network regions^1^**

Secondary somatosensory cortex (left)	-60	-12	3	10.3	10431	3.20E-42	✓
Primary somatosensory cortex (left)*	-54	-11	37	8.87	-	-	✓
Primary auditory cortex (right)	54	0	-11	9.22	6709	5.31E-32	
Secondary somatosensory cortex (right)*	51	-31	18	8.1	-	-	✓
Cerebellum posterior lobe (left)	-44	-79	-30	8.12	6346	6.50E-31	
Cerebellum posterior lobe (right)	23	-72	-23	9.63	5745	4.57E-29	
Premotor cortex (right)	48	1	44	8.29	2027	1.41E-15	
Thalamus (right)	4	-19	5	6.93	1221	1.01E-11	✓
Thalamus (left)*	-6	-23	4	6.82	-	-	✓
Prefrontal cortex (left)	-6	40	-27	9.87	930	3.96E-10	✓
Visual cortex V3 (left)	-30	-98	-15	7.22	560	5.96E-08	
Middle temporal gyrus (right)	61	-34	-2	5.69	366	2.03E-06	
Premotor cortex (left)	-15	13	61	7.18	296	7.39E-06	
Insular cortex (right)	38	11	1	6.97	196	5.84E-05	✓
Anterior caudate nuclei (left)	-21	25	0	6.49	159	0.00014	
Anterior caudate nuclei (right)	12	26	-2	6.01	119	0.00037	

Group results from FSL analysis. Cluster-threshold of Z ≥ 5 and p = 0.001. Decreasing cluster size order. MNI, Montreal Neurological Institute

^1^From Apkarian et al, 2005

*Local maxima within the above cluster

## Discussion

A method for dentoalveolar stimulation using a MR-compatible device was developed. The advantages of this device include: anatomical and functional MRI data acquisition without apparent image distortion, presence of a customizable bite bar that reduces head motion, MR-compatibility [22], modular design that fits within the restricted space between the subject's head and the radiofrequency (RF) coil, and ability to access all dentoalveolar quadrants.

Few fMRI studies using dentoalveolar stimulation are reported in the literature, likely due to the technical challenges associated [17]. This is illustrated by the approach of Miyamoto and colleagues, where an operator stimulated lower lip and tongue of subjects using a stick with a piece of Velcro at its tip and generated torque forces in the right upper incisor adding a rubber tip with a groove [14]. Three other fMRI dentoalveolar studies used somewhat more sophisticated stimuli delivery, using vibrotactile [15] or electrical stimulation of teeth [16,17]. Common to these stimulation methods were their fixed stimulation site and lack of more rigid head stabilization. The present device avoids these shortcomings by allowing reach of buccal oral tissues in all intraoral quadrants, and by providing a customizable bite fork that is comfortable for the subjects and significantly reduces head motion during MRI data acquisition.

Dynamic pressure was the stimulus of choice since a significant number of AO patients describe increased sensitivity to touch at the intraoral pain site, mentioning that they avoid chewing around that area - a feature that it is not well described in the literature [6,7,23,24] with recent quantitative sensory testing evidence supporting this clinical finding [8]. Our psychophysical results provided additional support to this given the sensitivity, specificity and discriminant ability values found for the two groups when using such stimulus. Our pilot data suggests that the stimulus device can potentially discriminate AO subjects from painfree controls using dynamic pressure pain stimuli.

In testing the stimulus device during fMRI data acquisition, we devised a simple experimental design with a blocked stimulation paradigm that is known to give robust results with high statistical power and relatively large BOLD signal changes [25], but it is more susceptible to anticipation, habituation and attention modulatory effects [18]. Another simplification due to our reduced sample size was the use of fixed-effects group-level analysis, which uses a within-subject variance that prevents generalization of our findings to the population level [26]. It is important to highlight that the fMRI results reported here are a preliminary proof-of-concept for the stimulus device feasibility to be used in an MR environment. Different factors could potentially explain the mean group brain activations found in our small sample, and this limited pilot study was not designed to parse out those factors' influence; therefore little can be said on these activations meaning. Furthermore, the presence of the lip/cheek retractor could potentially add a modulatory effect to the results that is not fully understood. Taking these caveats into consideration, our fMRI results are in agreement with those of a meta-analysis that reported a network of brain areas that are activated during acute pain stimulation in painfree subjects [21]. FMRI data collection using the same protocol as described here is ongoing on both painfree controls and AO subjects, and it may provide us a better picture of brain activation following dentoalveolar dynamic pressure in healthy and diseased states, as there is evidence that these may be different [21].

Limitations encountered were long assembly time prior to MR imaging (up to 15 minutes), which may be of importance given the costs involved for scanner time, and need for previously trained operators for device assembly and fitting. With practice, the assembly time can be decreased and with further development of our device it can be both streamlined and broaden in its scope. Modifying this device to support other auxiliary modules, such as intraoral thermal probe, Von Frey filaments or to be fit in RF head coils with diverse geometry, are a few possibilities to accommodate different research needs. Regarding fMRI research specifically, more reliable stimulus delivery can be achieved by using a computer to trigger stimulus on- and off-set, what has been suggested as a way of reducing BOLD signal changes variability and to increase sensitivity for brain activation [19]. This putative versatility may help serve a growing need to better measure sensory functions of the human trigeminal system [20,27] by adding a way to map intraoral somatosensory representation in the central nervous system.

## Conclusions

A novel device that delivers dentoalveolar dynamic pressure stimulation was developed and validated. It allows investigators to deliver this and potentially other psychophysical modalities in all quadrants of the oral cavity, and, importantly, its MR-compatibility provides an opportunity to correlate dental chair-side psychophysical findings with MRI-acquired data.

## Methods

### Intraoral stimulus device

The intraoral stimulus device was developed to deliver dynamic pressure over the subject's gingiva and/or oral mucosa so that future research can evaluate the differences between subjects of interest, in this case AO patients, and painfree controls. The device, capable of providing a range of stimuli, would be within the RF head coil during MRI data acquisition; therefore its components should meet the most stringent MR-compatibility classification, so no detectable magnetic forces or torque is imposed on the device and there is negligible or no image distortion [22]. The device constituent and ancillary parts are: supporting frame, bite fork, montage tools, intraoral probe and cam, knob and pivoting joint, and lips and cheeks retractor (Figure 1). For a comprehensive description of the stimulus device, see additional file 1: Appendix - Dentoalveolar Stimulus Device Description.

#### Laboratory force measurements

The relationship between the force delivered by the intraoral probe in grams and the number of elastic bands was measured with a strain gauge attached to the probe. A voltmeter, specialized software for resistance readings (LJstreams, LabJack Corporation, Lakewood, CO) and a set of standardized weights tied to the intraoral probe tip were used to estimate the pressure delivered by the intraoral probe according to the number of 3.175 mm (1/8") elastic bands used. After assembling the elastic band(s), the voltmeter was reset and weights increments were added until there was a noticeable movement of the probe and the resistance readings reached a plateau, which meant that weight addition would provoke probe translation but minimal voltmeter reading changes. The weight that first elicited probe translation was interpreted as the minimal force the intraoral probe tip exerted when using a particular number of 3.175 mm (1/8") elastic band(s). Two trials for each amount of elastic band, from 1 to 8 elastic bands, were performed.

### Subjects

Atypical odontalgia patients were recruited from those seeking care from Dr. Donald Nixdorf at the University of Minnesota Temporomandibular Disorders, Orofacial Pain and Oral Medicine clinic. Inclusion criteria for these AO patients were:

•Presence of intraoral pain with the following characteristics:

∘Localized in a endodontically treated tooth or in a place formerly occupied by a tooth (gingiva, oral mucosa, alveolar bone);

∘Present for more than 6 months;

∘Non-paroxysmal in character, and present for eight hours or more within a 24-hour period;

∘Can be provoked/increased by applying pressure to the intraoral site;

•No signs of gross pathology present during clinical examination or in available radiographic imaging.

These criteria are in accordance with recent studies involving AO subjects [8,10,24]. Control subjects were recruited from the University of Minnesota community. Inclusion criterion for age- and gender-matched controls was absence of intraoral pain in the previous six months. Exclusion criteria for both groups were presence of the following conditions, as determined by history and physical exam:

•Tooth pathology, sinus infection, trigeminal neuralgia, herpes zoster;

•History of destructive trigeminal nerve procedures or trauma-associated facial bone fractures within the trigeminal nerve distribution;

•Migraine headache, cluster headache or paroxysmal hemicrania;

•Pregnancy, planning pregnancy or the potential of being pregnant; and

•Claustrophobia.

Telephone or in-person screening was performed to assess subject eligibility criteria fit. The Institutional Review Board of the University of Minnesota approved the study protocol, and all subjects participating provided informed consent.

### Study protocol

Subjects were scheduled for three experimental visits, two at dental chair-side (cases and controls) and the last in the MR scanner (controls only). Two data points were collected during the first visit (initial and 1-hour pressure pain thresholds), while in the second visit we measured the 1-week pressure pain threshold and collected pain ratings during blocked-design stimulation. This psychophysical data was used to evaluate the reliability/reproducibility, sensitivity, specificity, discriminant ability, and validity of the device. In the third visit, that included only painfree controls, we tested its MR-compatibility by visual inspection of the images produced and the stimulus evoked-brain activation through statistical analysis of functional brain images.

#### First visit - Initial and 1-hour dynamic pressure pain threshold

Initially subjects received explanation about the study protocol and gave their informed consent. Bite impression was taken using an elastic material (Express™ bite, 3 M ESPE^®^, St Paul, MN) over the bite fork. Use of a Computerized Visual Analog Scale (COVAS, Medoc^® ^Ltd, Ramat Yishai, Israel) for pain scoring was explained, where 0 meant no pain and 10 the worst pain imaginable. After stimulus device fitting, the subject was asked to record specific pain scores without stimulation to ensure that they could produce the ratings that were intended.

The stimuli were delivered over the reported dentoalveolar pain site in cases and to a matched location in controls, at a frequency of approximately 1 Hz starting at a light force using one 3.175 mm (1/8") elastic band. According to the subject's pain rating, the pressure was increased by adding more elastic bands until one of the two possible endpoints occurred: a) consistent pain rating within 3-5 out of 10 using the COVAS or b) maximum pressure was reached (= 8 elastic bands).

**Figure 6:**
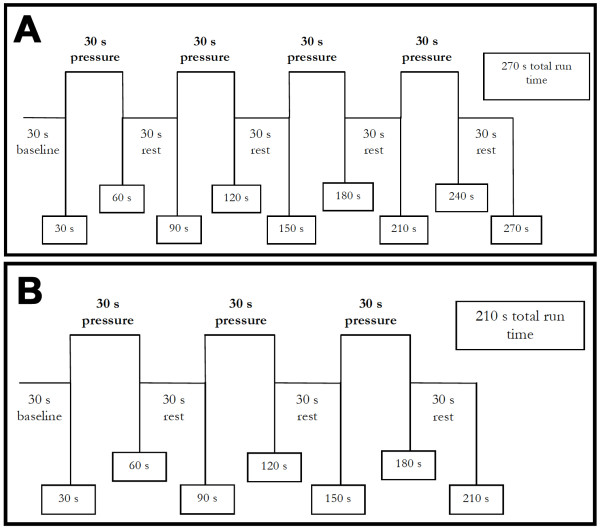
**Blocked design stimulus**. (A) depicts the stimulus provided in the dental chair-side setting, while (B) depicts the stimulus during fMRI data acquisition.

After a 1-hour rest period, the stimulus device was re-positioned so that the intraoral probe tip was over the same dentoalveolar location and adjusted to a pressure one level below the pain threshold. After confirmation or readjustment of the pressure to reach either endpoint, one run with four 30 s ON/OFF blocks preceded by a 30 s baseline was done, totaling 270 s (Figure 6A). This blocked-design run was delivered as training, since the second visit would include 4 repetitions of this run for all subjects during which pain ratings would be collected.

#### Second visit - 1-week dynamic pressure pain threshold and full stimulus protocol

Seven to ten days after the initial visit, the subject was accommodated in the dental chair with the stimulus device assembled to reach the same dentoalveolar location as during the first visit. The pressure was also initiated one level below the last threshold found, and it was adjusted as necessary to reach one of the two outcomes described for the first visit.

Once the threshold was re-established, four runs using the same block design protocol as the first visit were performed (Figure 6A). This procedure had three goals: assess if the intraoral stimuli could consistently elicit pain ratings within the target range (3-5 out of 10), acquaint painfree subjects with the stimulus protocol that would take place during fMRI data acquisition, and also to determine if the subjects were able to endure a full session of experimental intraoral pain. Although only painfree controls were planned to undergo the fMRI session for the present pilot study, an expansion of this study is planned which would include imaging data acquisition for AO cases. Therefore, such expansion justified acquainting all subjects with the stimuli protocol for the fMRI visit.

#### Third Visit - Functional magnetic resonance imaging

**Figure 7:**
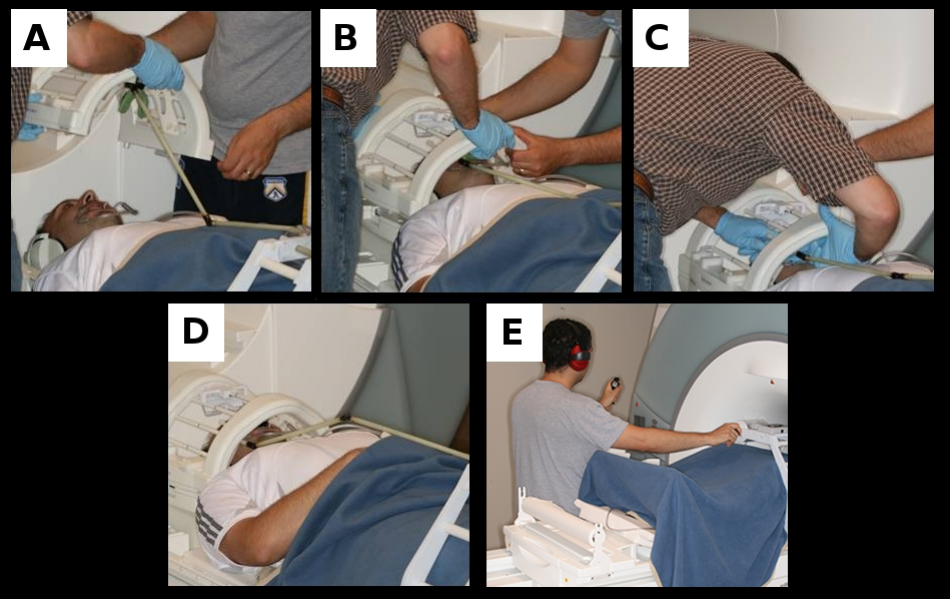
**Stimulus device assembly sequence for an fMRI session**. (A) depicts the lip retractor in place and the stimulator attached to the superior aspect of the RF head coil. (B) demonstrates the two-person technique in seating the stimulator in place with (C) depicting the need for repositioning components of the device when in place to achieve maximal comfort. (D) shows the stimulator device in position with the connector attached and supported. (E) depicts operation of the device with the subject's head positioned at the isocenter of the MRI scanner. Note: images were not taken from an optimal position due to safety precautions observed when in the proximity of the static magnetic field and subject gave consent to publish images.

Only painfree controls participated in this visit. The subject was received at the Center for Magnetic Resonance Research (CMRR) at the University of Minnesota, and an explanation of the MRI session was given and potential risks were discussed. A 3 Tesla Siemens Trio MR scanner with a circular polarized RF head coil was used. After placing the subject in the MR scanner bed, stimulus device fitting followed. The initial step was to position the lips and cheeks retractor in the subject. The superior part of the RF head coil was fitted since it had the supporting frame and intraoral components attached to it including the bite bar (Figure 7A-C), and the number of 3.175 mm (1/8") elastic bands used was that established as 1-week threshold (Table 2). Dentoalveolar location for stimulus delivery was the same as that during dental-chair visits. The framework supports the mechanism within the limited space between the RF coil and face of the subject (Figure 7D). Of note, during all image acquisition subjects were instructed to keep on biting on the bite bar, which held their individualized bite impression. This would not only allow the intraoral probe to stay at the same location throughout fMRI data acquisition but also it would minimize head motion. An arch with a knob was fitted over the scanner bed at the level of the subject's knees and this knob was then attached to a long stick connected to the cam device, in a way that a 90° knob rotation elicited full intraoral probe movement (Figure 7E). The final adjustment was to position the intraoral probe to barely touch the subject's dentoalveolar tissues over the stimulation site. Before imaging started, the subject underwent 5-10 seconds of dentoalveolar stimulation as to confirm pressure pain threshold and if needed, device fitting and/or number of elastic bands adjustment would take place to ensure pain stimulation at the same level as that found during the second visit full stimulus protocol.

After establishing communication with the subject via intercom, the imaging sequence protocol started. We first acquired T1-weighted magnetization prepared rapid gradient echo (MPRAGE) anatomical images (TR = 2530 ms, TE = 3.68 ms, flip angle = 7°, 224 axial slices, matrix 256 × 256, field of view = 245.76 mm^2^, voxel size = 0.96 × 0.96 × 1 mm^3^). Then four to six functional runs were performed using T2*-weighted echo-planar imaging (TR = 3000 ms, TE = 30 ms, flip angle = 90°, 36 axial slices, 70 volumes, matrix 64 × 64, field of view = 192 mm^2^, voxel size = 3 × 3 × 5.625 mm^3^) while intraoral stimulation was triggered by an operator inside the magnet room using a similar blocked design as during the dental chair visits but with only 3 on/off-blocks, totaling 210 s (Figure 6B). The beginning of the functional run was visually signaled by the operator in the control room to the second operator inside the magnet room, while the timing of stimulus delivery for the remainder of the functional run was controlled by the latter using a digital stopwatch to mark block on/offset. At the end of the imaging session the subject was removed from the magnet bore and a brief inspection of intraoral tissues took place. We asked the subject to do an overall pain rating for the imaging session in a 0 to 10 numerical scale. This completed the subject participation in the study.

#### Imaging data processing and analysis

FMRI data processing was carried out using FMRIB's Expert Analysis Tool (FEAT) 5.98, part of FSL 4.1.4 (FMRIB's Software Library, http://www.fmrib.ox.ac.uk/fsl). Anatomical and functional images were skull-stripped using FSL's brain extraction tool [28]. The first three volumes were discarded from each functional run due to elevated MR signal prior to reaching a longitudinal magnetization steady state. Visual inspection of functional images in cine mode was used to detect gross head movements (> 3 mm in any direction). Pre-analysis processing steps included motion correction using FMRIB's Linear Image Registration Tool (FLIRT) [29], interleaved slice timing correction, spatial smoothing using a 5 mm full-width-at-half-maximum (FWHM) kernel, grand-mean intensity normalization, and temporal highpass filtering (cutoff 60 s) to remove low-frequency noise. Co-registration of functional to structural images was done with FLIRT, and they were then normalized to the Montreal Neurological Institute (MNI) 152 brain template at 1 mm^3 ^resolution using FLIRT. Registration of anatomical images to the MNI template was further refined using FMRIB's Nonlinear Image Registration (FNIRT) tool with a 10 mm warp resolution.

Following preprocessing, each functional run analysis was carried out using FMRIB's Improved Linear Model (FILM) with local autocorrelation correction [30]. Explanatory variable (EV) for dentoalveolar dynamic pressure pain stimulation was modeled with a boxcar function, and an EV for the temporal derivative of stimulation timing was generated. The EVs were then convolved with a double-gamma hemodynamic response function. Correction for multiple comparisons was done using cluster-based thresholding of Z statistic images using a cluster significance threshold of Z ≥ 2.3 and p = 0.05. Functional runs for each subject were averaged using FEAT fixed-effects model. Group-level analysis also used FEAT fixed-effects model with a more stringent cluster thresholding (Z ≥ 5, p = 0.001). Threshold activation maps were then overlaid on the group structural mean image to define anatomical location of activations by using five atlases available in FSLview display tool: Harvard-Oxford cortical and subcortical, Juelich histological, MNI structural and Tailarach.

Additionally, three-dimensional digital models of each subject's brain were created using Freesurfer image analysis suite 4.0.5 http://surfer.nmr.mgh.harvard.edu/[31,32]. FEAT output of each non-spatially smoothed functional run was registered to the respective subject's brain model, and this registration allowed the subject-level FEAT output to be resampled to the MNI 305 template cortical surface using a surface-based spatial smoothing of 5 mm FWHM. Surface-based group analysis was done using fixed-effects, one-sample group mean model similar to the group-level analysis done with FSL. False discovery rate at 0.05 threshold was used for multiple comparisons correction [33].

## Abbreviations

AO: atypical odontalgia; MR: magnetic resonance; MRI: magnetic resonance imaging; fMRI: functional magnetic resonance imaging; RF: radiofrequency; TR: time to repeat; TE: echo time; CMRR: Center for Magnetic Resonance Research; BOLD: blood-oxygen-level-dependent; EPI: echo planar imaging; COVAS: computerized visual analog scale; ICC: interclass correlation coefficient; CI: confidence interval; ANOVA: analysis of variance; ROC: receiver operating characteristic; MPRAGE: magnetization prepared rapid gradient echo; FMRIB: Oxford centre for Functional Magnetic Resonance Imaging of the Brain; FSL: FMRIB Software Library; MNI: Montreal Neurological Institute; EV: explanatory variable.

FWHM: full-width-at-half-maximum; FLIRT: FMRIB's Linear Image Registration Tool; FNIRT: FMRIB's Nonlinear Image Registration; FILM: FMRIB's Improved Linear Model; FEAT: FMRIB's Expert Analysis Tool.

## Authors' contributions

DRN and EJM conceptualized the research. EJM, DRN and NH designed the device. DRN, EJM and NH designed the study protocol, with input from DAB and MTJ. DRN and EJM recruited subjects and collected the data, which included pain ratings, laboratory calibration, dental chair-side assessment and reliability testing, and MRI scanning. EJM and MTJ analyzed the data, with input from DRN and NH. EJM and DRN interpreted the data, with input from NH, MJT and DAB. EJM wrote the manuscript with the assistance of DRN, MTJ, DAB and NH. EJM and DRN wrote the response to reviewer comments and made changes in the manuscript, with input from NH, MJT and DAB. All authors read and approved the final manuscript.

## Supplementary Material

Additional file 1 **Appendix - Dentoalveolar Stimulus Device Description**. Comprehensive description of the stimulus device and its constituent materials. A diagrammatic scheme of the device is also included. Click here for file
