# Supplementary material for: Evaluation of a magnetic resonance-compatible dentoalveolar tactile stimulus device
Source: BMC Neurosci. 2010 Oct 28;11:142. doi: 10.1186/1471-2202-11-142 (PMC2988799; doi:10.1186/1471-2202-11-142)
Supplement: Additional file 1 — Appendix - Dentoalveolar Stimulus Device Description. Comprehensive description of the stimulus device and its constituent materials. A diagrammatic scheme of the device is also included. [file 1471-2202-11-142-S1.PDF]

APPENDIX

DENTOALVEOLAR STIMULUS DEVICE DESCRIPTION

The stimulus device provides a mean to deliver sensory stimulation to a subject's gingiva and/or oral mucosa overlying the buccal aspect of maxillary and mandibular dentoalveolar processes with a specially designed probe. Stimuli can be delivered both at dental chair-side and also during MR imaging. All parts of the stimulus device are made of plastic material, thus guaranteeing its MR-compatibility. Please see figure A1 for a diagrammatic view of the stimulus device fitted inside an example of head coil for MRI (CP head coil).

The intraoral probe is made of autoclavable plastic with a curved-axis and small head area. The curved-axis helps avoid touching the subject's labial commissure during stimulation while the reduced head size propitiates an oral mucosa/gingival contact area of approximately 2 mm^2. The probe is made to move in a vertical plane and impinge obliquely against a small segment of the dentoalveolar process during the stimulation cycle. The reciprocating motion is created by the action of a rotating cam on a pivot linkage supporting the probe, and is thus naturally cyclical and limited in amplitude (5-7 mm excursion). The force of contact between the probe and the dentoalveolar process is limited to the force supplied by elastic bands in the linkage. The subject is fitted with a custom plastic lip and cheek retractor to expose the dentoalveolar processes to the probe, so that it only touches those processes avoiding the labial commissure and internal surfaces of cheeks. The probe, its supporting linkage, and the actuating cam are mounted on a small (approx. 75 x 25 x 5 mm) base plate, which is movable over a limited range to reach all four dentoalveolar quadrants. The probe's base plate is clamped by a screw knob to a larger arch-shaped vertical plate, which fits closely inside the CP head coil during MR imaging, while a 3-piece metallic structure is used to support the base plate during dental chair-side use.

During MR imaging the arch plate (approx. 250 x 120 x 7mm) is mounted to the top half of the CP head coil using the rails provided on the coil for its head clamp. The arch can be clamped at various positions along the rail to align the probe to the subject's dentoalveolar processes. The arch plate also supports a plastic bite bar, which can be covered with quick-set silicone putty holding the subject's bite impression. The subject will grip the bite bar with his teeth to stabilize the position of the dentoalveolar processes to the probe system. A second, much larger arch-shaped structure (approx. 500 x 300 x 50mm) will be attached to the MR scanner bed at the position of the subject's knees. This structure will support the investigator's end of the manual control rod used to turn the actuating cam. The arch will span the scanner bed and be fastened to the edges of the table and held in place by the table's straps.

The manual control rod will telescope to adjust its length to a given setup. The input end of the rod will be turned manually by the investigator. It is fitted with an indicator knob to show rotary position (stimulus on/off position).

MATERIALS

Contact probe	Kel-F PCTFE
Bite fork	glass filled polycarbonate
Probe linkage<br>Cam<br>Probe base plate<br>Arch plate<br>Arch plate clamps	Delrin copolymer acetal
Screws	Nylon
Linkage pivot pin	Vespel PI
Elastic bands	Latex
Control rod	G10 glass filled epoxy
Rod support arch structure	White PVC type1
Lip retractor	Acrylic

We are willing to share further details of our device, as well as our experience developing it for this environment. For those interested, please address the corresponding author, Dr. Donald Nixdorf (nixdorf@umn.edu).

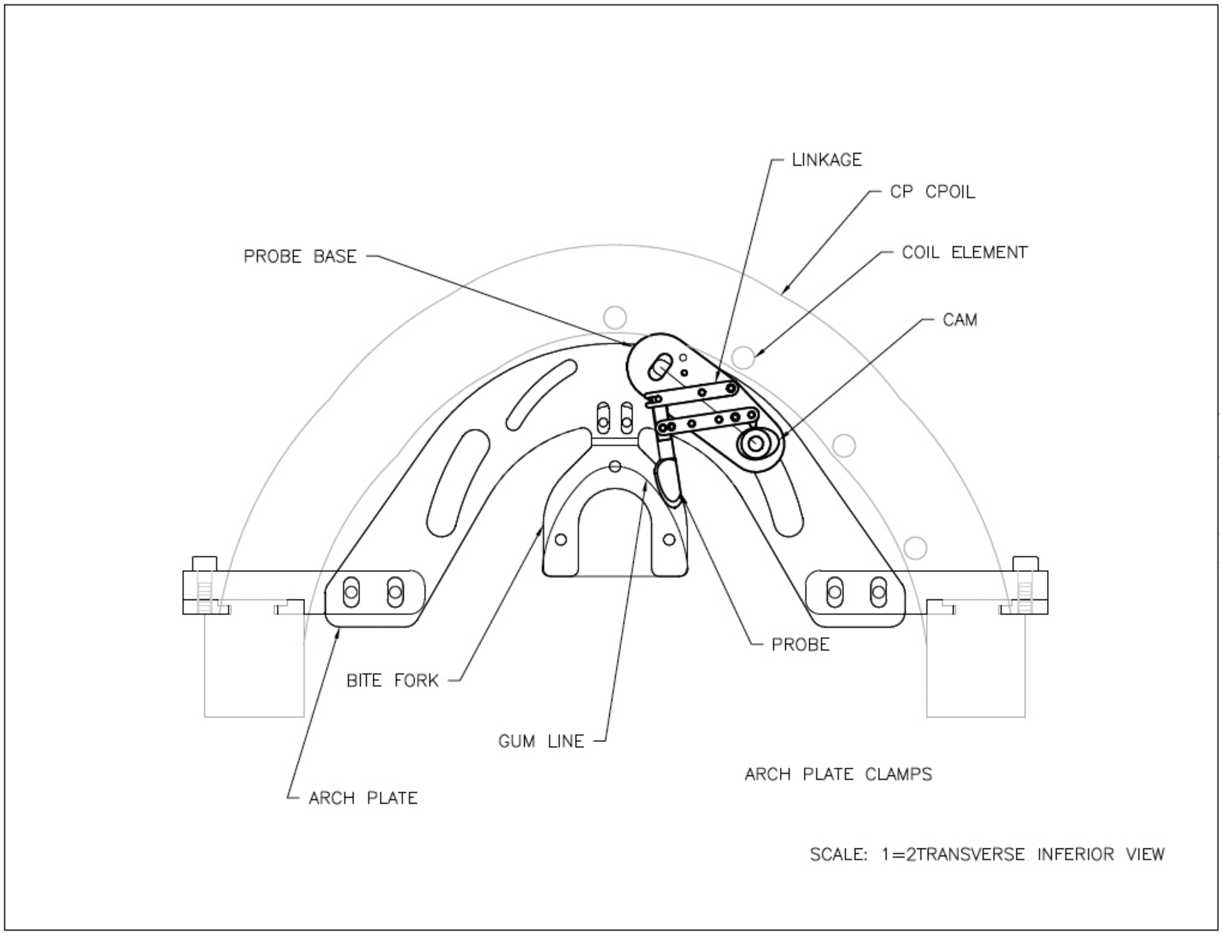

Figure A1 – Stimulus device diagram
